# Supplementary material for: Metabolic Dependency Shapes Bivalent Antiviral Response in Host Cells in Response to Poly:IC: The Role of Glutamine
Source: Viruses. 2024 Aug 30;16(9):1391. doi: 10.3390/v16091391 (PMC11436187; doi:10.3390/v16091391)
Supplement: Supplementary file 1 [file viruses-16-01391-s001.zip › viruses-3145894-supplementary.pdf]

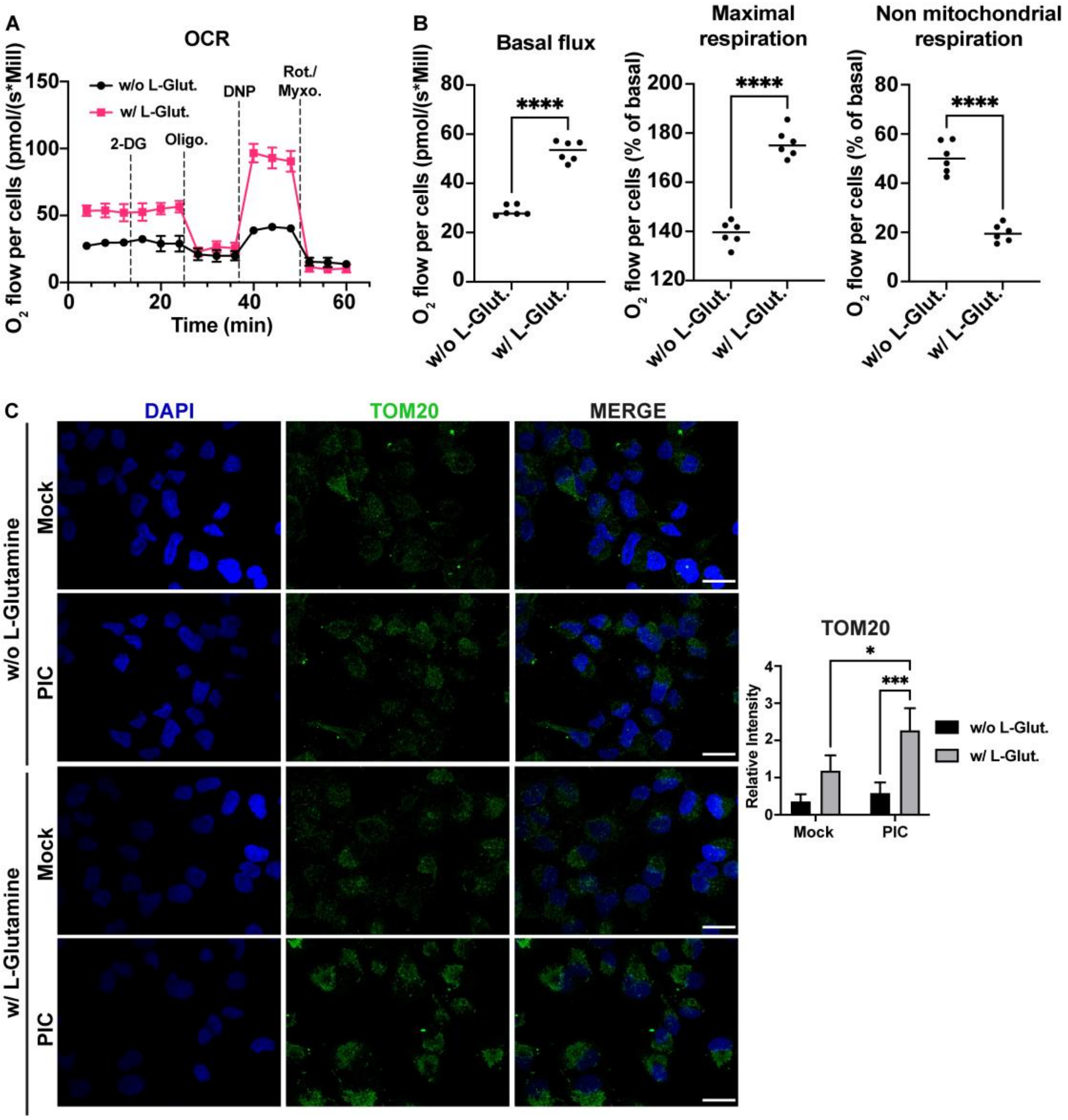

Figure S1. OXPHOS and mitochondrial functionality are increased in response to poly:IC. (**A**) Comparison of the oxygen consumption rate of A549[Dual] cultured with or without L-glutamine and stimulated with poly:IC. 5 mM 2-deoxyglucose (2-DG) has been used to inhibit hexokinase activity and glycolysis. 1 µM oligomycin (Oligo) has been used to inhibit ATP synthase. 100 µM 2,4-dinitrophenol (DNP) has been used as protonophoric uncoupler, allowing maximal respiration. 1 µM Rotenone – 1 µM Myxothiazol has been used to uncouple both mitochondrial complex I and III, illustrating non mitochondrial respiration. Poly:IC stimulation induces a higher rate of OXPHOS in cells grown in RPMI supplemented with L-glutamine than cells cultured without L-glutamine. (**B**) More specifically, cells cultured with L-glutamine have higher basal O_2 consumption flux, associated with higher maximal respiration capacity and consistently lower non mitochondrial respiration, in response to poly:IC stimulation. Error bars represent standard deviation of three independent experiments. **** p<0.0001. (**C**) TOM20 expression was assessed by immunofluorescence in A549[Dual], grown in RPMI 2 g·L^{-1} glucose, supplemented or not with 2 mM, using PIC 0.25 µg·mL^{-1}. These images are representative of three independent experiments. Scale bar: 10 µm. Error bars represent standard deviation from three independent experiments. * p<0.0332; *** p<0.0002.

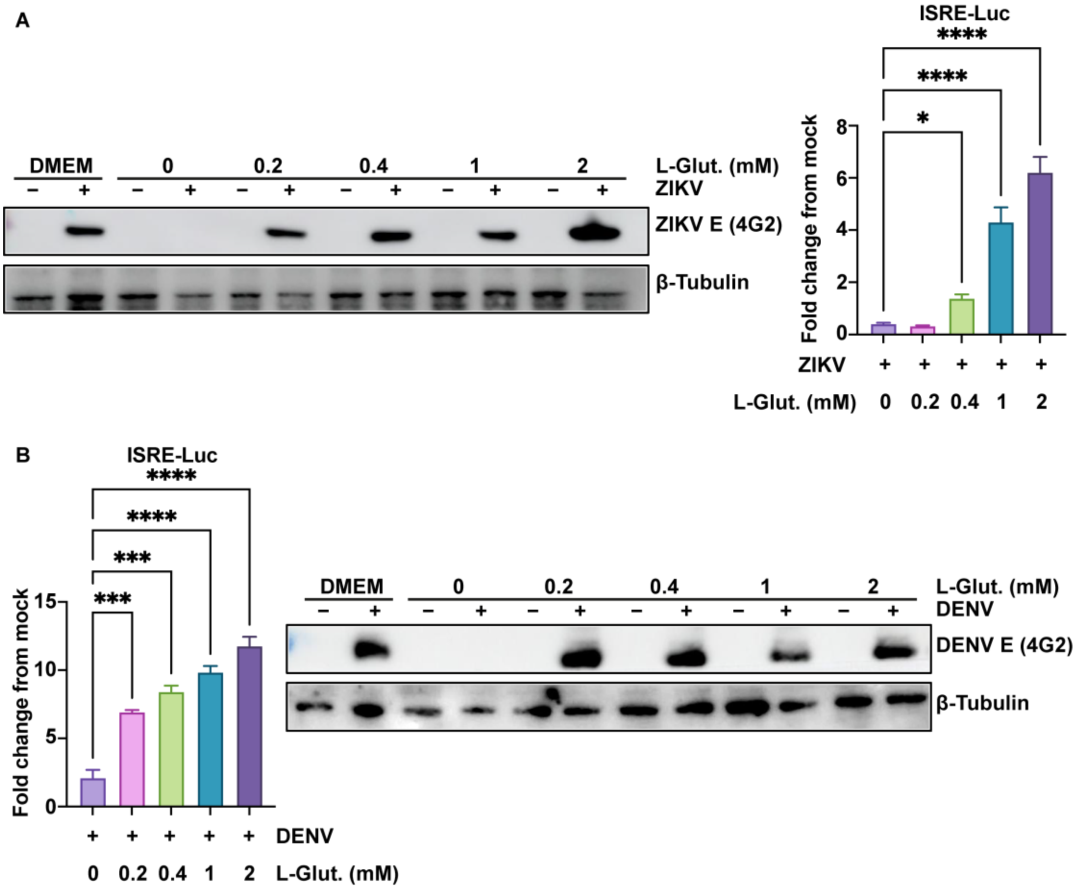

Figure S2. Effect of glutamine on the establishment of the antiviral response during flaviviral infection. A549^{Dual} grown in RPMI 2 $g{\cdot}L^{-1}$ glucose, supplemented or not with 2 mM L-glutamine, were infected with Zika Virus (**A**) and Dengue Virus (**B**) at MOI 2 respectively during 48h and 24h. The infection was confirmed by immunodetection of the viral envelope protein (E) using a pan-flaviviral anti-E antibody (mouse 4G2). These images are representative of three independent experiments. Evaluation of IRF pathway activation in response to infection was achieved by measuring activity of secreted Lucia luciferase, using QUANTI-Luc substrate. Results are expressed as fold change in emitted luminescence intensity from mock infected cells. Error bars represent standard deviation from three independent experiments. * p<0.0332; ** p<0.0021; *** p<0.0002; **** p<0.0001.

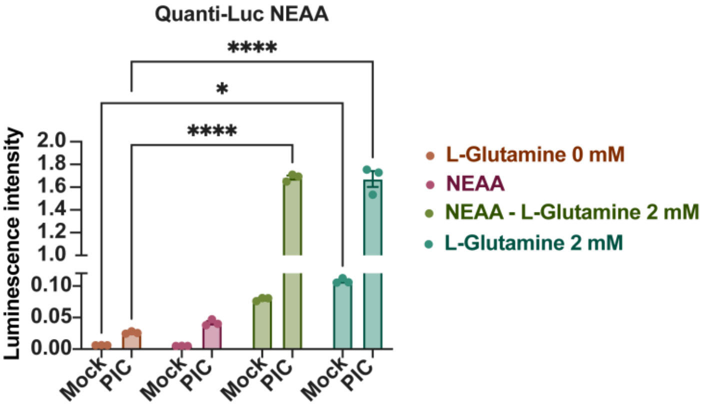

Figure S3. Antiviral response upregulation is independent of non-essential amino acids. Evaluation of IRF pathway activation in response to PIC 0.25 $\mu g{\cdot}mL^{-1}$ stimulation of A549^{Dual} grown in RPMI 2 $g{\cdot}L^{-1}$ glucose supplemented with either or both non-essential amino acids – NEAA and 2 mM L-glutamine, was achieved by measuring activity of secreted Lucia luciferase, using QUANTI-Luc substrate 24 hours after treatment. Results are expressed as emitted luminescence intensity. Error bars represent standard deviation of three independent experiments. *p<0.0332; ** p<0.0021; *** p<0.0002; **** p<0.0001.

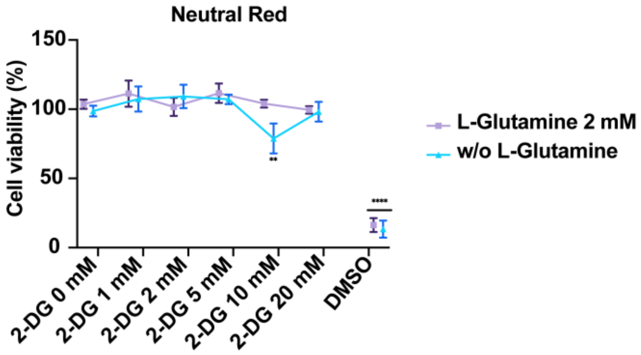

Figure S4. 2-deoxyglucose exerts no cytotoxic effect. We treated A549[Dual] with 2-deoxyglucose with concentrations ranging from 1 mM to 20 mM for 24 hours. We then evaluated the impact of these different concentrations on cell viability by neutral red assay.
